# Study on the Effects and Mechanisms of Sea Buckthorn Fruit Pulp in Improving Polycystic Ovary Syndrome

**DOI:** 10.1002/fsn3.72181

**Published:** 2026-08-12

**Authors:** Han Xiaoya, Yang Yuanyuan, Peng Qingjie, Chen Xiaojiang, Ma Cunling, Li Na, Tian Xiaofan, Wu Xin, Liu Hetao, He Rui

**Affiliations:** ^1^ Key Laboratory of Fertility Maintenance, Ministry of Education Ningxia Medical University Yinchuan China; ^2^ College of Traditional Chinese Medicine Ningxia Medical University Yinchuan China; ^3^ Reproductive Center of General Hospital Ningxia Medical University Yinchuan China; ^4^ Tianjin Institute of Urology The Second Hospital of Tianjin Medical University Tianjin China

**Keywords:** PI3K/AKT/mTOR, polycystic ovary syndrome, sea buckthorn, sea buckthorn pulp

## Abstract

Polycystic ovary syndrome (PCOS) is a common endocrine and metabolic disorder characterized by ovarian dysfunction, hyperandrogenism, and insulin resistance. This study investigated the therapeutic effects and potential mechanisms of sea buckthorn pulp (Pu; *Hippophae rhamnoides* fruit pulp) in a letrozole‐induced PCOS mouse model. PCOS‐like mice were established by letrozole administration combined with a high‐fat diet and subsequently treated with Pu or metformin for 39 days. Body weight, glucose metabolism, estrous cyclicity, ovarian histopathology, and serum hormone levels were evaluated. Network pharmacology and Western blot analyses were performed to explore the potential molecular mechanisms. Pu treatment significantly reduced body weight gain and improved glucose tolerance and insulin sensitivity in PCOS mice (*p* < 0.05). In addition, Pu restored estrous cyclicity, reduced the numbers of cystic and atretic follicles, and improved ovarian morphology. Serum testosterone and luteinizing hormone levels were significantly decreased, whereas estradiol levels were partially restored compared with the untreated PCOS group (*p* < 0.05). Pu treatment was also associated with increased ovarian expression of p‐PI3K and p‐mTOR. These findings suggest that sea buckthorn pulp ameliorates reproductive and metabolic dysfunction in PCOS mice, and its therapeutic effects may be associated with modulation of the PI3K/AKT/mTOR signaling pathway. Further studies are warranted to confirm the underlying mechanisms and evaluate its translational potential in clinical settings.

## Graphical abstract

Sea buckthorn pulp improves metabolic and reproductive dysfunction in a letrozole‐induced PCOS mouse model and is associated with modulation of the PI3K/AKT/mTOR signaling pathway.

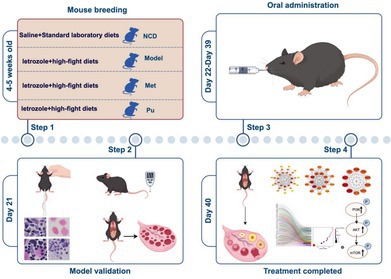

## 1 Introduction

Polycystic ovary syndrome (PCOS) is a common reproductive endocrine disorder among women of childbearing age (Stener‐Victorin et al. 2024), with a prevalence ranging from 10% to 13% (Liu, Jiang, et al. 2024). PCOS is characterized by hyperandrogenism, oligo‐ or anovulation, and polycystic ovarian morphology and is frequently accompanied by insulin resistance, chronic low‐grade inflammation, and metabolic dysfunction (Zhang, Chen, and Zhao 2025). These reproductive and metabolic abnormalities seriously affect female fertility and long‐term health.

Current pharmacological treatments for PCOS mainly focus on symptom management. Combined oral contraceptives (COCs) are recommended for improving hyperandrogenism and menstrual irregularities in adult women with PCOS (Zhang, Huang, et al. 2025). However, COCs have limited efficacy in improving infertility and polycystic ovarian morphology and may increase the risk of adverse effects during long‐term use, especially in patients with metabolic disorders (Hoeger et al. 2021; Liu, Xiao, et al. 2024). Although hormonal therapy and surgery can alleviate certain symptoms, the pathogenesis of PCOS remains incompletely understood, and recurrence after treatment is common (Liu, Xiao, et al. 2024). Therefore, safer and more effective therapeutic strategies for PCOS are still needed.

Traditional Chinese Medicine (TCM) has been widely applied in the treatment of gynecological disorders, including PCOS (Chen, Deng, et al. 2023). Sea buckthorn (*Hippophae rhamnoides L*.), a medicinal and edible plant, has attracted increasing attention because of its multiple biological activities (Pharmacopoeia Commission of the People's Republic of China (2020)). Phytochemical studies have shown that sea buckthorn contains abundant bioactive compounds, including flavonoids, phenolic compounds, tannins, amino acids, sterols, and organic acids (Wang, Zhao, et al. 2022). Among these compounds, quercetin, kaempferol, and isorhamnetin are considered major active constituents with antioxidant and metabolic regulatory activities (González‐Arceo et al. 2023). Previous studies have demonstrated that sea buckthorn exhibits antioxidant, anti‐inflammatory, anti‐diabetic, lipid‐lowering, hepatoprotective, and gut microbiota‐modulating effects and may improve obesity‐related metabolic disorders (Chen, Cai, et al. 2023; Nybom et al. 2023; Żuchowski 2023). Sea buckthorn pulp, produced by physically pressing ripe sea buckthorn fruits, retains abundant bioactive constituents and possesses antioxidant and hypoglycemic activities (Ding et al. 2022). Preliminary clinical observations from our research group suggested that sea buckthorn pulp may improve menstrual irregularities and hyperandrogenism‐related symptoms in patients with PCOS. However, its therapeutic effects and underlying molecular mechanisms in PCOS remain unclear.

The PI3K/AKT/mTOR signaling pathway plays an important role in glucose metabolism, follicular development, and ovarian cell proliferation, and its dysregulation has been implicated in the pathogenesis of PCOS. Therefore, modulation of this pathway may represent a promising therapeutic strategy for PCOS.

In the present study, network pharmacology combined with GO and KEGG enrichment analyses was used to predict the potential targets and signaling pathways of sea buckthorn pulp in PCOS. A letrozole‐ and high‐fat diet‐induced PCOS mouse model was subsequently established to evaluate the effects of sea buckthorn pulp on metabolic dysfunction, ovarian morphology, and reproductive abnormalities. Furthermore, the involvement of the PI3K/AKT/mTOR signaling pathway was experimentally validated to elucidate the underlying therapeutic mechanisms.

## 2 Materials and Methods

### 2.1 Materials

#### 2.1.1 Animals

A total of 30 female C57BL/6J mice, aged 4–5 weeks and weighing approximately 20 g, were used in this study. The mice were of Specific Pathogen Free (SPF) grade and obtained from the Experimental Animal Center of Ningxia Medical University (Animal Ethics Approval No.: IACUC‐NYLAC‐2023‐137; Animal Facility License No.: SYXK (Ning) 2020–0001). The animals were housed under controlled environmental conditions, with a constant temperature (20°C–22°C), relative humidity (60%–80%), and a 12‐h light/dark cycle. Food and water were provided ad libitum. Prior to the formal experiment, all mice underwent a 1‐week acclimation period under the same standard conditions. Eligible individuals were subsequently selected based on the regularity of their estrous cycles.

The study protocol was approved by the Animal Welfare and Ethics Committee of Ningxia Medical University. All procedures involving animals were conducted in strict accordance with the approved protocol and the relevant guidelines for animal welfare and ethics.

#### 2.1.2 Main Reagents

Letrozole tablets (Jiangsu Hengrui Pharmaceuticals Co. Ltd., specification: 2.5 mg) and metformin hydrochloride tablets (Sino‐American Shanghai Squibb Pharmaceuticals Co. Ltd., specification: 0.5 g) were purchased from the General Hospital of Ningxia Medical University. Sea buckthorn pulp was provided by Gansu Longyuanhong Biotechnology Co. Ltd. (Test No.: THW 0123–0142; Sample No.: TH‐YP20240421‐001). The preparation process involved using mature sea buckthorn fruits as raw material, which were washed, crushed, pulped, and filtered to yield a natural pulp that retains the fruit flesh and part of the bioactive components. The pulp primarily contains vitamins, flavonoids, polysaccharides, unsaturated fatty acids, organic acids, and various trace elements. Based on this, we performed metabolomic sequencing on the sea buckthorn pulp (Table S1). The dosage of sea buckthorn pulp used in this study was determined based on preliminary clinical observations conducted by our research group (unpublished observations), preliminary animal experiments, and body surface area normalization according to the “Equivalent Dose Ratio Table for Conversion between Human and Animal Body Surface Areas.” (Nair and Jacob 2016) Based on these considerations, the human‐equivalent dose was converted to the corresponding mouse dose, and 2.5 mL/kg/day was selected as the standard treatment dose. The solution was freshly prepared before each administration. The solution was freshly prepared prior to each administration. The BCA protein quantification kit was purchased from Kaiji Biotechnology Co. Ltd., and Western blot reagents were obtained from Biyuntian Biotechnology Research Institute. Fluorescent secondary antibodies were acquired from Abbkine Co. Ltd., China. Rabbit anti‐β‐actin antibody was purchased from ABclonal, and rabbit anti‐DDX4/MVH antibody was obtained from Abcam. Rabbit anti‐mTOR, rabbit anti‐p‐mTOR, rabbit anti‐p‐PI3K, rabbit anti‐AKT, rabbit anti‐S6, and rabbit anti‐p‐S6 antibodies were all purchased from Cell Signaling Technology. Rabbit anti‐PI3K antibody was purchased from Shanghai Yamei Biopharmaceutical Technology Co. Ltd., and rabbit anti‐p‐AKT antibody was obtained from Affinity Biosciences. High‐fat diet (60% fat content) purchased from Research Diets, item number D12492.

#### 2.1.3 Instrument

Confocal laser microscope (Nikon, Japan); BX‐51 upright fluorescence microscope (OLYMPUS, Japan); GelDoc XR System chemiluminescent gel imaging system purchased from Bio‐Rad Instruments, USA.

### 2.2 Methods

#### 2.2.1 Establishment and Treatment of PCOS Animal Models

A total of 30 female C57BL/6J mice were used in this study. After 1 week of acclimatization (during which all mice were fed a standard chow), they were randomly divided into a normal control diet group (NCD, *n* = 9) and a model group (Model, *n* = 21) using a random number table. First, following an established classic PCOS modeling protocol, a PCOS model was induced by daily oral gavage of 1 mg/kg letrozole (dissolved in normal saline) for 21 days (Zhang, Huang, et al. 2025). The normal control group received an equal volume of normal saline via gavage. During the modeling period, body weight and fasting blood glucose levels were monitored weekly. Starting from day 14 of modeling, vaginal smears were collected daily for seven consecutive days to assess the estrous cycle. The estrous cycle stages were determined based on cytological characteristics: proestrus (predominantly nucleated epithelial cells), estrus (predominantly cornified epithelial cells), metestrus (mixture of cornified epithelial cells and leukocytes), and diestrus (predominantly leukocytes) (Li et al. 2025). Successful model establishment was indicated by estrous cycle disruption, specifically the absence of the estrus stage and persistent diestrus. After the modeling period, oral glucose tolerance tests (OGTT) and insulin tolerance tests (ITT) were performed. Following model validation, three mice from the control group and three from the model group were euthanized to confirm successful PCOS induction.

Subsequently, the remaining 18 successfully modeled mice were randomly divided into three groups (*n* = 6 per group) using a random number table: the model group (Model), the positive drug control group (Met), and the sea buckthorn pulp group (Pu). The Met group received metformin hydrochloride at a dose of 200 mg/kg/day via oral gavage (Wang, Wang, et al. 2022). The Pu group received sea buckthorn pulp at a dose of 2.5 mL/kg/day via oral gavage. Both the normal control group (NCD) and the Model group received an equivalent volume of normal saline via gavage. The treatment period lasted for 39 days. During the treatment period, body weight and fasting blood glucose were monitored weekly. After 21 days of treatment, the estrous cycle was continuously monitored for 7 days via vaginal cytology. At the end of the treatment period, oral glucose tolerance tests (OGTT) and insulin tolerance tests (ITT) were performed. Subsequently, euthanasia was performed on the mice using 1% pentobarbital sodium, followed by the collection of serum and bilateral ovaries. The body weight and the weights of both ovaries were measured. The left ovary was fixed in 4% paraformaldehyde, while the right ovary and serum were stored at −80°C for subsequent experiments.

#### 2.2.2 Vaginal Smear Test to Determine the Estrous Cycle

The estrous cycle was monitored by daily vaginal smear collection. Collections began 7 days after PCOS model establishment and continued for 9 days prior to terminal sampling, with all procedures performed at 4:00 PM daily. To obtain a smear, approximately 10 μL of 0.9% saline was gently introduced into the vaginal cavity using a Pasteur pipette and subsequently expelled onto a glass slide. Smears were examined under a light microscope immediately after preparation to determine the estrous cycle stage. Subsequently, representative smears from each experimental group were selected and stained using a commercial ultrafast, non‐toxic modified Papanicolaou (PAP) staining kit, strictly adhering to the manufacturer's protocol. Following staining, cell types were identified based on their coloration: nucleated epithelial cells (blue‐green), anucleated keratinized cells (pink), and leukocytes (blue‐purple) (Liang et al. 2024).

#### 2.2.3 Oral Glucose Tolerance Test (OGTT)

On day 19 of modeling, an oral glucose tolerance test (OGTT) was conducted via tail vein blood sampling. After 14–16 h of fasting (water provided ad libitum) and cage bedding renewal, a glucose solution (5 g in 1.2 mL 0.9% saline) was prepared by heating in a 100°C water bath until complete dissolution. Mouse body weight was measured to calculate individual gavage volume based on 3 g glucose per kg body weight. Following a small tail vein incision, fasting blood glucose was measured using a Roche glucometer. Mice were then orally administered the glucose solution, and blood glucose levels were recorded at 15, 30, 60, and 120 min post‐gavage. After the test, a normal diet was resumed. Blood glucose fluctuations were compared across experimental groups.

#### 2.2.4 Insulin Tolerance Test (ITT)

An insulin tolerance test (ITT) was performed on day 21 of modeling via tail vein blood sampling. After 4–6 h of fasting (water provided ad libitum) and cage renewal, a glucose rescue solution (5 g in 1.2 mL 0.9% saline) was prepared by heating in a 100°C water bath. Mouse body weight was measured to calculate the individual insulin dose (1 IU/kg). Following a small tail vein incision, fasting blood glucose was measured using a Roche glucometer. Insulin was then administered intraperitoneally, and blood glucose levels were recorded at 15, 30, 60, and 120 min post‐injection. After the test, the normal diet was resumed. Blood glucose fluctuations were compared across experimental groups.

#### 2.2.5 Mice Weight Gain Rate

Body weight was measured at the time of sacrifice. The rate of weight gain was determined according to the following formula: (Final weight−Initial weight)/Initial weight × 100%.

#### 2.2.6 Ovary H&E (Hematoxylin and Eosin) Staining

For morphological analysis, ovarian tissues were processed as follows: after 24‐h fixation in 4% paraformaldehyde at room temperature, they were dehydrated, paraffin‐embedded, and sectioned at 4 μm. The resulting sections were then stained with hematoxylin and eosin (H&E) and examined under an OLYMPUS BX‐51 upright fluorescence microscope to assess pathological changes in ovarian structure.

#### 2.2.7 Determination of Serum Hormone Levels

Following collection, cardiac blood was centrifuged (4000 rpm, 10 min, 4°C) to obtain serum. Serum concentrations of sex hormone‐binding globulin (SHBG), follicle‐stimulating hormone (FSH), luteotropic hormone (LH), estradiol (E2), testosterone (T), and progesterone (PROG) were determined by ELISA according to the manufacturer's instructions.

#### 2.2.8 Immunofluorescence

Following deparaffinization, 4‐μm ovarian tissue sections underwent antigen retrieval in citrate buffer. After blocking endogenous peroxidase and permeabilizing with 0.5% Triton X‐100, sections were incubated overnight with rabbit anti‐DDX4/Mvh antibody (1:400). Following washes, sections were incubated with a fluorophore‐conjugated secondary antibody (1:300) in the dark. Nuclei were counterstained with DAPI. Imaging was performed on a Nikon confocal microscope, with DDX4/Mvh (green, cytoplasmic) and DAPI (blue, nuclear) signals captured.

#### 2.2.9 EdU Assessment

Cell proliferation was detected using BeyoClick EdU‐488 (Beyotime, China). EdU was prepared in PBS at a concentration of 100 mg/kg, and the prepared EdU was intraperitoneally injected into mice 4 h before euthanasia. The procedure was carried out according to the manufacturer's instructions. After EdU staining, four random fields were captured under a fluorescence microscope.

#### 2.2.10 Network Pharmacology

First, using the TCMSP database (https://old.tcmsp‐e.com/tcmsp.php), we screened the active components of sea buckthorn (SJ), and after removing duplicate data, obtained the corresponding drug targets. Next, use the PubChem database (https://pubchem.ncbi.nlm.nih.gov/) in combination with the Swiss Target website (http://swisstargetprediction.ch/index.php) to predict potential targets. Subsequently, search for PCOS‐related targets separately using the keywords “PCOS” and “polycysticovarysyndrome” in the GeneCards (https://www.genecards.org/), OMIM (https://www.omim.org/), and DisGeNET (https://www.disgenet.org/) databases. After removing duplicates, the resulting list represents the disease targets for PCOS. Using the Venny 2.1.0 website (https://bioinfogp.cnb.csic.es/tools/venny/), a Venn diagram was created to illustrate the overlap between the targets of sea buckthorn and those associated with polycystic ovary syndrome. Subsequently, we employed Cytoscape 3.9.1 software to construct a network diagram of sea buckthorn active components and their targets.

The GEO database (https://www.ncbi.nlm.nih.gov/geo/) collects a vast amount of clinical and experimental data, which can serve as a clinical relevance anchor for animal experiments. Using “PCOS” as the keyword and restricting the species to “ *Homo sapiens* ,” we performed a search and obtained the dataset file GSE48301. This dataset is a well‐annotated, publicly available resource derived from human ovarian granulosa cells of PCOS patients and matched controls, making it directly relevant to ovarian pathophysiology (Cui et al. 2025). It is widely cited in PCOS transcriptomic studies, ensuring data reliability (Miao et al. 2022). The dataset includes six cases of overweight/obese PCOS (according to NIH criteria) and six overweight/obese controls. Microarray analysis was performed on fluorescence‐activated cell sorting (FACS)‐isolated endometrial epithelial cells (eEP), endothelial cells (eEN), stromal fibroblasts (eSF), and mesenchymal stem cells (eMSC). Using the online analysis tool GEO2R, the gene chip dataset was divided into normal and PCOS groups, with differential expression genes defined as those with a *p*‐value < 0.05 and |log_2_FC| ≥ 1. Genes with log_2_FC ≥ 1 were defined as up‐regulated, and those with log_2_FC ≤ −1 as down‐regulated. Volcano plots were generated using the online platform bioinformatics.com.cn, and Venn diagrams were constructed to compare the targets of SJ with the up‐regulated and down‐regulated genes from GSE48301, respectively. The GEO database (https://www.ncbi.nlm.nih.gov/geo/) collects a substantial amount of clinical and experimental data. We performed a search using the keyword “PCOS” and the species “ *Homo sapiens* .” We obtained the GSE48301 dataset, which includes samples from six overweight/obese PCOS patients (NIH criteria) and six overweight/obese controls. Microarray analysis was conducted on fluorescence‐activated cell sorting (FACS)‐isolated endometrial epithelial cells (eEP), endothelial cells (eEN), stromal fibroblasts (eSF), and mesenchymal stem cells (eMSC). Using the GEO2R online analysis tool (https://www.ncbi.nlm.nih.gov/geo/geo2r/), the gene chip datasets were divided into normal and PCOS groups, with a *p*‐value < 0.05 and |log_2_FC| ≥ 1 set as the threshold for differentially expressed genes. Genes with log_2_FC ≥ 1 were defined as upregulated, while those with log_2_FC ≤ −1 were defined as downregulated. Volcano plots were generated using the Bioinformatics Online Platform (http://www.bioinformatics.com.cn/), and Venn diagrams were constructed to compare the targets of SJ with the upregulated and downregulated genes in GSE48301.

Draw a truncated diagram of PCOS genes, GEO datasets, and SJ targets in GraphPad Prism 10.0. Use the bioinformatics platform to find the intersection between SJ targets and the upregulated and downregulated genes in GSE48301, presenting it in the form of a Venn diagram. Additionally, download the GPL file from GEO and process the GSE48301 data to select the top 40 intersecting targets, presenting them as a heatmap to analyze their expression differences. Then, sort and display the top 10 upregulated and downregulated genes based on log_2_FC values, and use the online bioinformatics platform to create bar charts.

We imported the intersection obtained from three databases into the STRING database (https://cn.string‐db.org/), set “Organisms” to “homo,” and obtained the target network map of SJ treating polycystic ovary syndrome. The resulting TSV file was imported into Cytoscape 3.9.1 and Cytoscape 3.7.0 software to construct the core target network map. Using the parameters Betweenness (BC), Closeness (CC), and Degree (DC) in the “CytoNCA” plugin, data calculation was performed. Core targets were screened with Degree ≥ 15, followed by visual analysis, and the Degree values of the core targets were presented in a bar chart. Next, the Expression Analysis module on the GEPIA2 website (http://gepia2.cancer‐pku.cn/#index) was used to calculate correlations for hub genes. Finally, a heatmap of intra‐group correlations was drawn using the ChiPlot website (https://www.chiplot.online/).

Further enrichment analysis was conducted on the core targets of SJ in the treatment of polycystic ovary syndrome. Differential genes were imported into the DAVID website (https://david.ncifcrf.gov/), with the identifier set as “Officialgene symbol” and the species chosen as “Homo.” Biological process (BP), molecular function (MF), and cellular component (CC) entries, as well as KEGG pathway data, were downloaded separately. Bubble plots and Sankey diagrams were drawn using the Bioinformatics Online website.

#### 2.2.11 Western Blotting

Total protein was extracted from ovarian tissues, quantified by BCA assay, and denatured. Equal amounts of protein were separated by SDS‐PAGE, transferred to PVDF membranes, and blocked with 5% non‐fat milk. The membranes were then incubated overnight at 4°C with specific primary antibodies (dilution 1:1000 for all targets, including β‐actin, PI3K, p‐PI3K, AKT, p‐AKT, S6, and p‐S6). After washing, HRP‐conjugated secondary antibodies were applied for 2 h at room temperature. Immunoreactive bands were detected by ECL and imaged using a GelDoc XR system, with subsequent quantification performed in ImageJ.

#### 2.2.12 Statistical Analysis

All data in this study were analyzed using GraphPad Prism software (version 10.0). Measurement data are expressed as mean ± standard deviation (*x̄* ± SD). Normality of data distribution was confirmed by the Shapiro–Wilk test, and homogeneity of variances was verified by Levene's test. Comparisons among multiple groups were performed using one‐way analysis of variance (One‐Way ANOVA). When a statistically significant difference was found, post hoc pairwise comparisons were conducted using Tukey's multiple comparisons test. All statistical tests were two‐tailed, and a *p*‐value of < 0.05 was considered statistically significant.

## 3 Results and Discussion

### 3.1 High‐Fat Feeding Combined With Letrozole Can Induce a PCOS Mice Model

**FIGURE 1:**
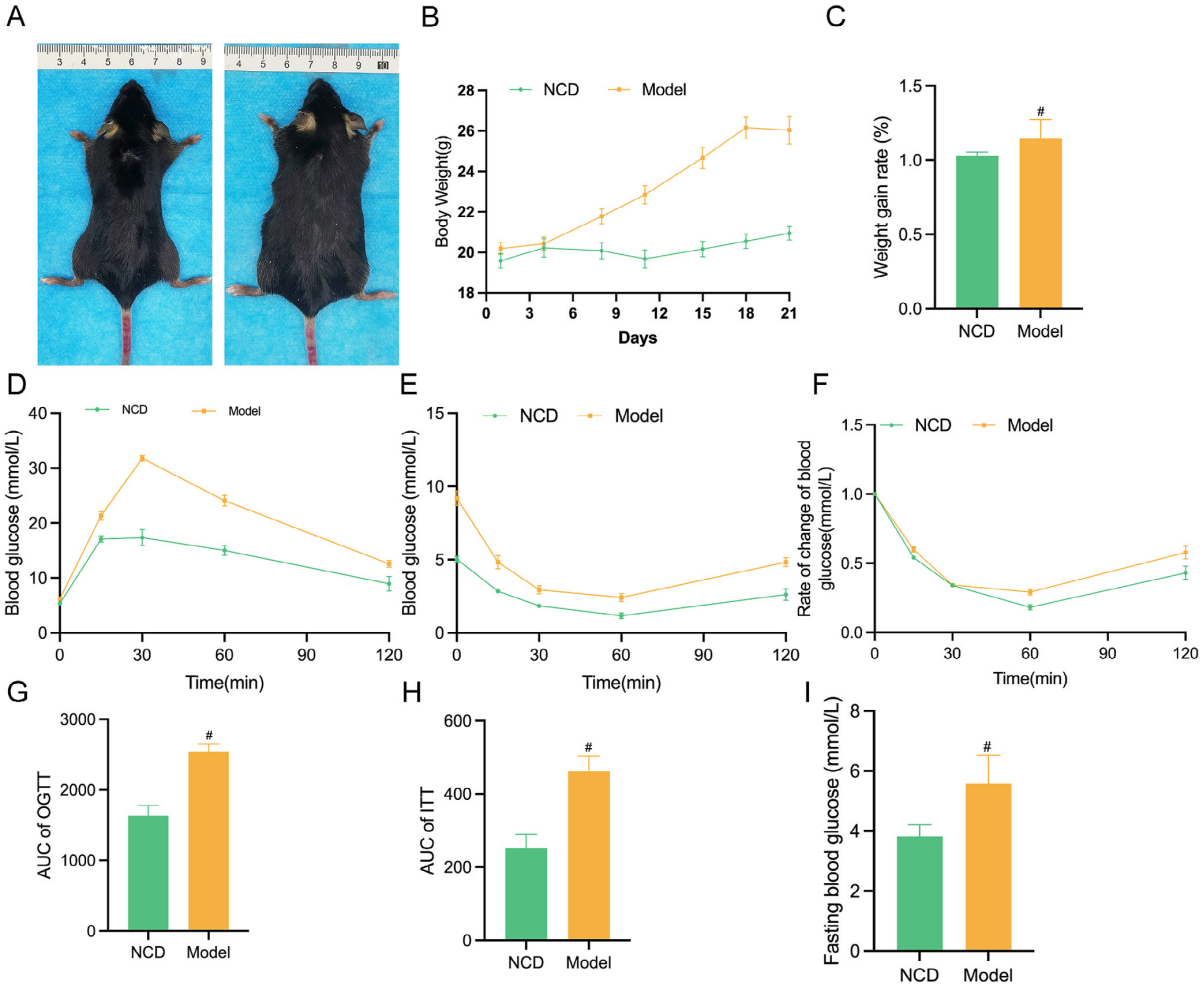
High‐fat diet combined with letrozole induces a mouse model of polycystic ovary syndrome. (A) Representative body photographs of mice in the normal control diet group (NCD) and the model group (Model); (B) Body weight change curves of NCD and Model group mice over the entire experimental period; (C) Body weight growth rate of NCD and Model group mice; (D) Oral glucose tolerance test (OGTT) curves of NCD and Model group mice; (E, F) Insulin tolerance test (ITT) curves; (G) Statistical analysis of the area under the curve (AUC) for OGTT; (H) Statistical analysis of the area under the curve (AUC) for ITT; (I) Comparison of fasting blood glucose levels between NCD and Model group mice. Data are presented as mean ± standard error of the mean (SEM), with *n* = 3 per group. The # symbol indicates a statistically significant difference compared to the normal control group, with *p* < 0.05.

**FIGURE 2:**
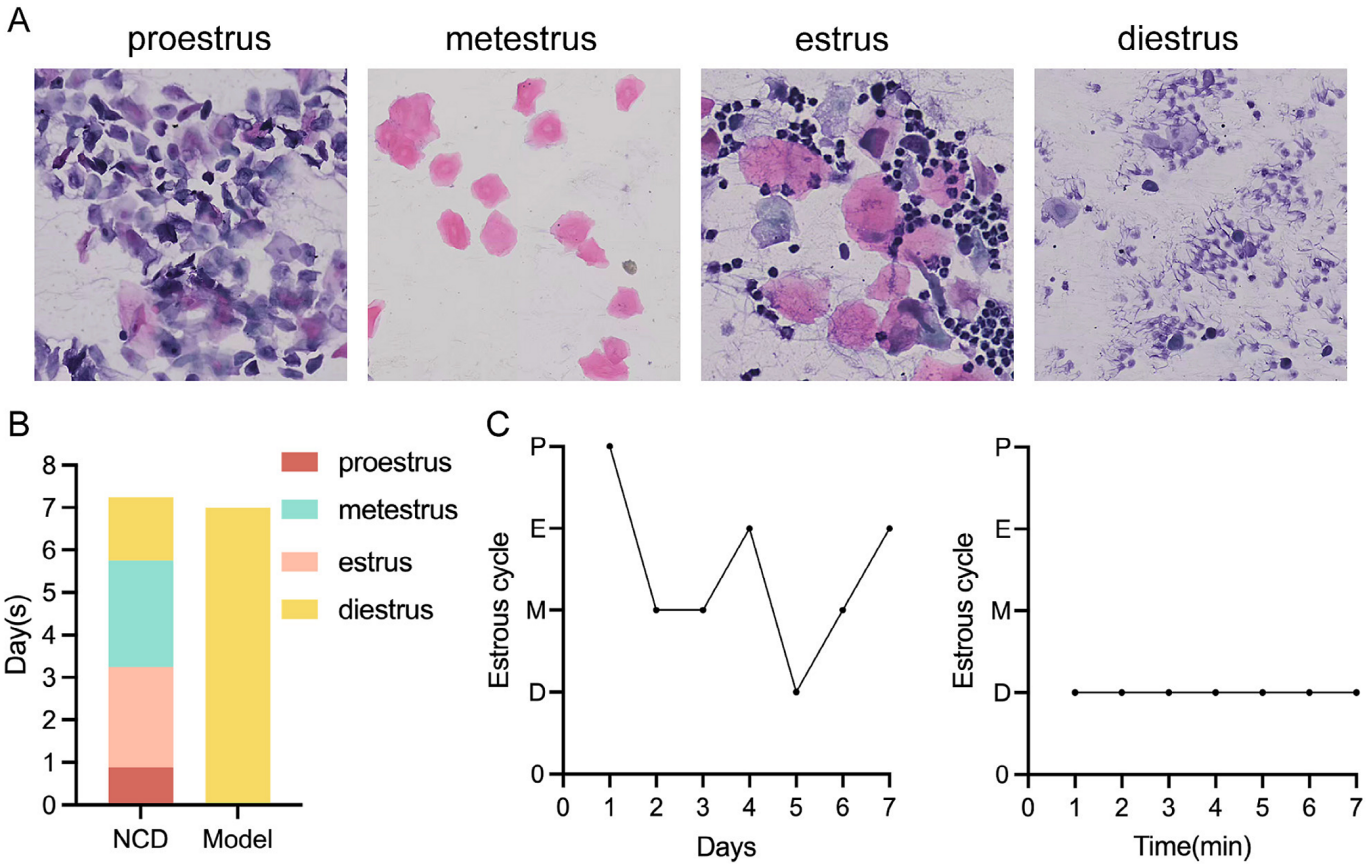
Disruption of the estrous cycle in PCOS model mice. (A) Staining of vaginal smears at different stages; (B) Distribution of the estrous cycle across groups; (C) Line graph illustrating the estrous cycle in different groups.

To establish a mouse model of polycystic ovary syndrome (PCOS), a combination of letrozole gavage and a high‐fat diet was administered for 21 days. Mice in the model group exhibited significantly accelerated body weight gain (Figure 1A–C), successfully simulating the metabolic obesity phenotype associated with PCOS. In terms of glucose metabolism, the model group displayed marked glucose intolerance, insulin resistance, and elevated fasting blood glucose levels (Figure 1D–I), suggesting the presence of metabolic dysfunction and insulin resistance characteristic of PCOS, thereby supporting the suitability of this model for subsequent investigations of metabolic improvement. Additionally, the estrous cycle of mice in the model group was completely arrested in the diestrus phase (Figure 2A–C), indicating successful replication of reproductive axis dysfunction. Taken together, these results demonstrate that the present study successfully established a classic PCOS mouse model exhibiting both metabolic abnormalities and reproductive cycle disorder.

### 3.2 Sea Buckthorn Fruit Pulp Exerts an Inhibitory Effect Against PCOS‐Like Phenotype in Rodent Models

**FIGURE 3:**
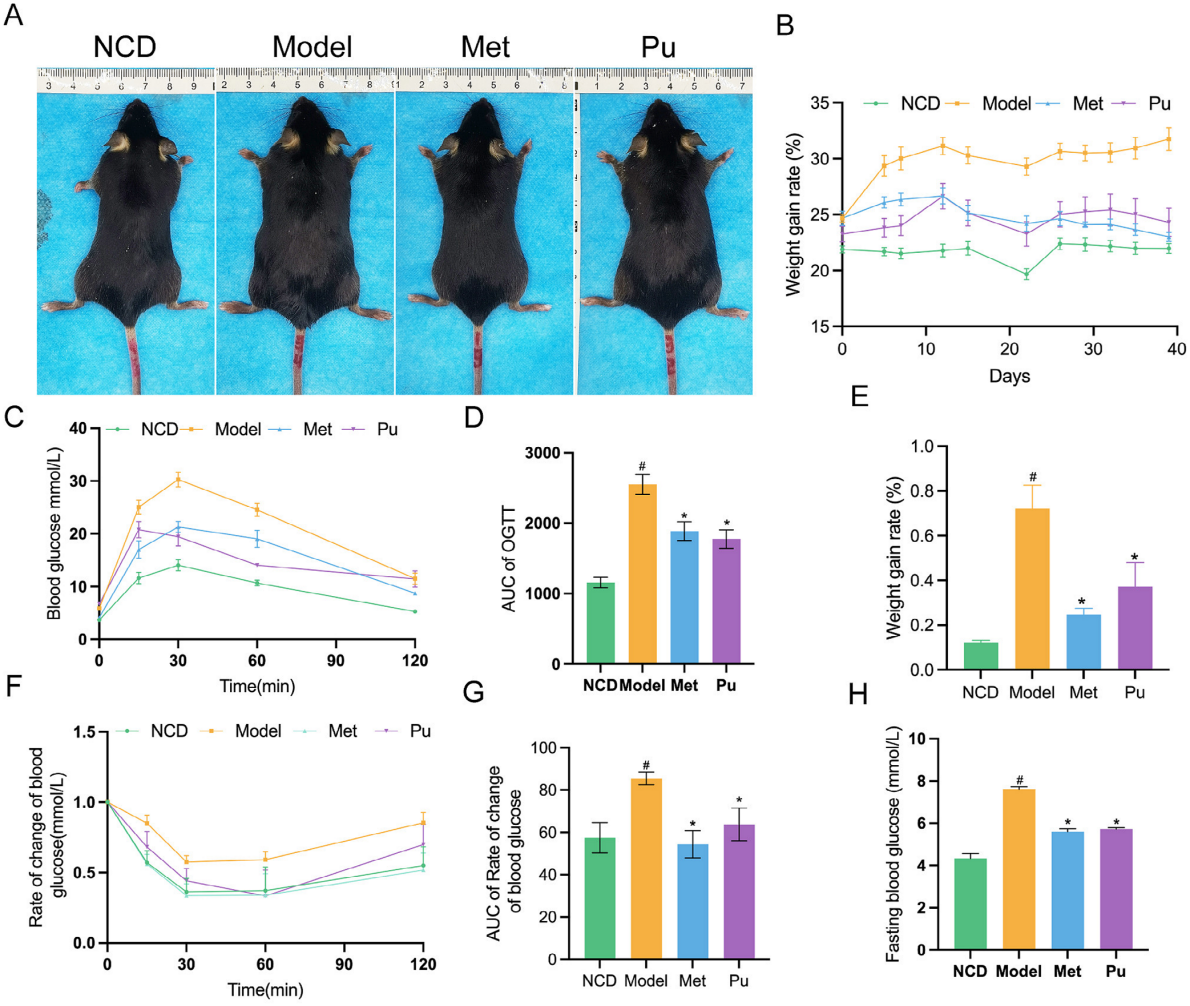
Sea buckthorn pulp ameliorates the metabolic phenotype in PCOS mice. (A) Representative body photographs of mice from each group at the end of the treatment period. (B) Body weight change curves of mice in each group during the treatment period. (C, D) Oral glucose tolerance test (OGTT) curves and the corresponding area under the curve (AUC) analysis. (E) Body weight change rate. (F, G) Insulin tolerance test (ITT) curves and statistical analysis of the area under the curve (AUC). (H) Fasting blood glucose levels in mice. Data are presented as mean ± standard error of the mean (SEM), with *n* = 6 per group. The symbol # indicates a statistically significant difference compared to the normal control group (*p* < 0.05), and the symbol * indicates a statistically significant difference compared to the model group (*p* < 0.05).

To clarify the therapeutic effects of sea buckthorn pulp, we examined its impact on the metabolic phenotype of PCOS mice. The results demonstrated that PCOS modeling led to accelerated body weight gain, whereas sea buckthorn pulp treatment effectively suppressed this trend, showing an effect comparable to that of metformin (Figure 3A,B,E). Further analysis of glucose metabolism revealed that sea buckthorn pulp significantly improved glucose intolerance and insulin resistance in PCOS mice, and also reduced random blood glucose levels (Figure 3C,D,F–H). Taken together, sea buckthorn pulp exhibited efficacy similar to that of the first‐line drug metformin in ameliorating PCOS‐related metabolic disturbances, such as obesity and insulin resistance. This finding not only validates its therapeutic potential but also suggests that its mechanism of action may involve targeting upstream components of insulin signaling or shared metabolic pathways.

### 3.3 Effects of Sea Buckthorn Pulp on Estrous Cycle and Serum Hormone Levels in PCOS Mice

**FIGURE 4:**
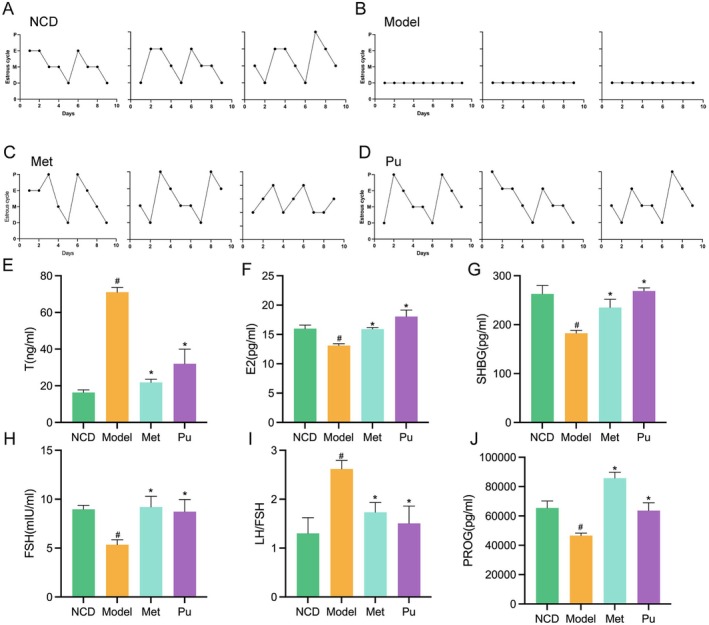
Sea buckthorn pulp ameliorates estrous cycle and sex hormone disturbances in PCOS mice. (A–D) Changes in the estrous cycle of mice in each group. (E) Comparison of serum testosterone (T) levels among groups. (F) Comparison of serum estradiol (E2) levels among groups. (G) Comparison of serum sex hormone‐binding globulin (SHBG) levels among groups. (H) Comparison of serum follicle‐stimulating hormone (FSH) levels among groups. (I) Comparison of the LH/FSH ratio among groups. (J) Comparison of serum progesterone (PROG) levels among groups. Data are presented as mean ± standard error of the mean (SEM), with *n* = 6 per group. The symbol # indicates a statistically significant difference compared to the normal control group (*p* < 0.05), and the symbol * indicates a statistically significant difference compared to the model group (*p* < 0.05).

After evaluating the improvement in metabolic phenotype by sea buckthorn pulp, we further investigated its regulatory effects on reproductive endocrinology and the estrous cycle. First, estrous cycle monitoring revealed that mice in the PCOS model group exhibited a complete arrest of the estrous cycle in the diestrus phase, indicative of a loss of cyclicity. In contrast, treatment with sea buckthorn pulp significantly promoted the restoration of regular estrous cyclicity, an effect comparable to that of metformin (Figure 4A–D). This directly demonstrates that sea buckthorn pulp not only ameliorates metabolic abnormalities but also reverses the core reproductive phenotype of PCOS—ovulatory dysfunction.

To investigate the underlying endocrine mechanisms, we measured serum sex hormone levels. Mice in the PCOS group exhibited elevated androgen levels (Figure 4E) and gonadotropin dysregulation (increased LH/FSH ratio, Figure 4I), accompanied by decreased levels of E2, FSH, SHBG, and PROG (Figure 4F–H,J). Intervention with sea buckthorn pulp effectively reversed these abnormalities, as evidenced by reduced T levels, a decreased LH/FSH ratio, and restored levels of E2, FSH, SHBG, and PROG (Figure 4E–J). Collectively, sea buckthorn pulp corrected hyperandrogenism and gonadotropin imbalance in PCOS mice, re‐establishing a sex hormone profile closer to normal. This comprehensive improvement in hormonal levels provides a direct endocrinological explanation for the observed restoration of the estrous cycle, suggesting that its therapeutic effect may be mediated through the modulation of hypothalamic–pituitary–ovarian (HPO) axis function.

### 3.4 Effects of Sea Buckthorn Pulp on Ovarian Morphology in PCOS Mice

**FIGURE 5:**
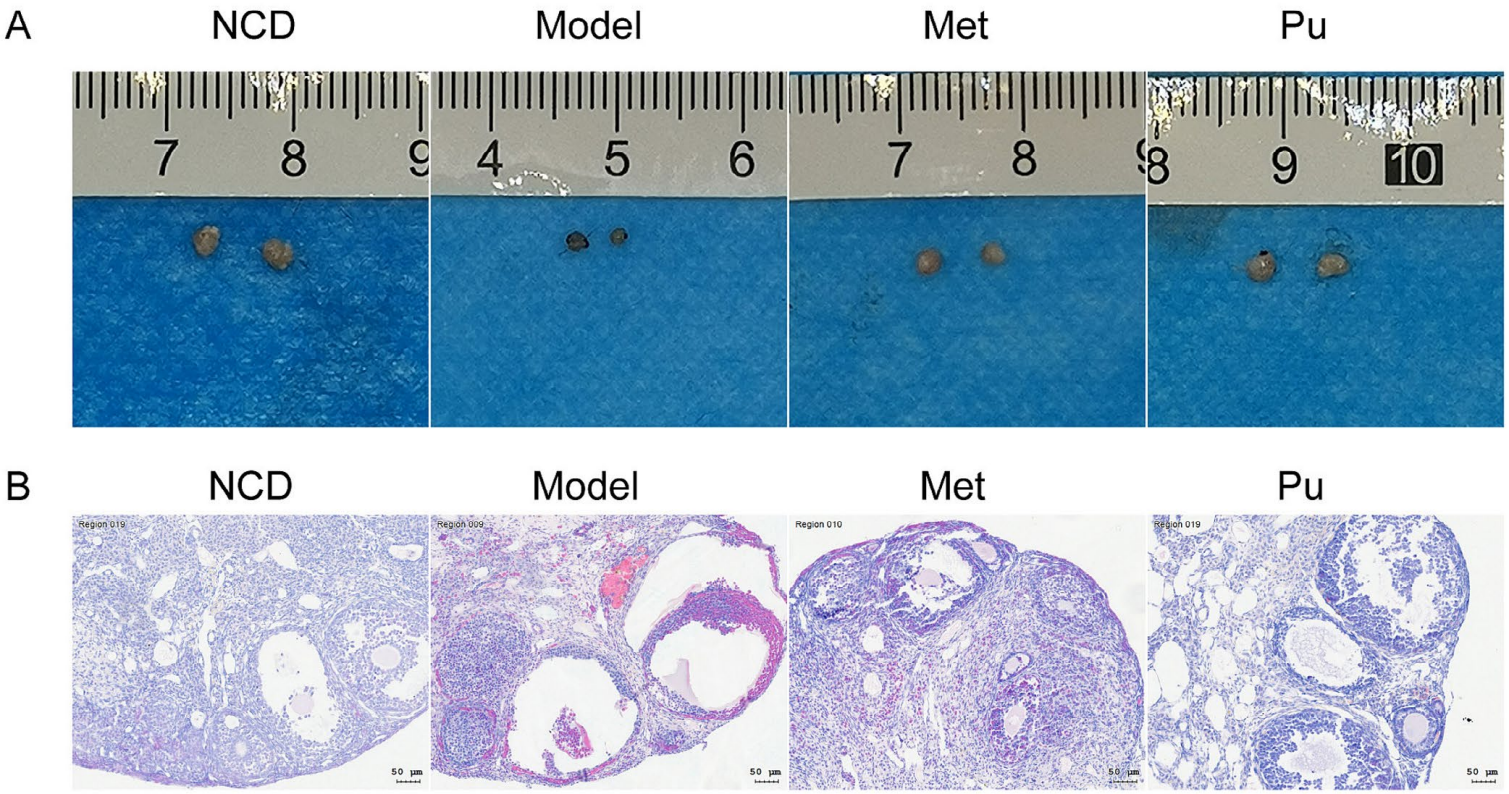
Sea buckthorn pulp improves ovarian morphology in PCOS mice. (A) Morphological comparison of ovaries from mice in each group. (B) Hematoxylin and eosin (H&E) staining of ovarian tissue. Scale bar: 50 μm. Data are presented as mean ± SEM, with *n* = 6 per group.

To evaluate the improving effect of sea buckthorn pulp on PCOS ovaries at the histological level, we examined ovarian morphology and performed H&E staining analysis. Macroscopically, the ovaries of mice in the PCOS model group appeared atrophic, whereas sea buckthorn pulp treatment restored their morphology (Figure 5A). H&E staining sections (Figure 5B) showed that the control group exhibited normal ovarian structure with well‐developed follicles at various stages and tightly arranged, orderly granulosa cell layers. In contrast, the model group displayed typical PCOS‐like pathological changes: a reduced total number of follicles, disorganized arrangement of granulosa cells, occasional absence of oocytes, and the presence of multiple cystically dilated follicles. Following sea buckthorn pulp treatment, the histological structure of the ovaries was significantly improved, as evidenced by a marked reduction in the number of cystic follicles, a more regular arrangement of granulosa cells, and clearer follicular structures at different developmental stages. These results provide direct histological evidence that sea buckthorn pulp not only improves the endocrine environment and cyclicity but also directly promotes the reversal of pathological ovarian structure. The restoration of granulosa cell arrangement suggests a potential recovery of their function, while the reduction in cystic follicles indicates effective suppression of abnormal follicular development.

### 3.5 Effects of Sea Buckthorn Pulp on Ovarian Proliferative Function

**FIGURE 6:**
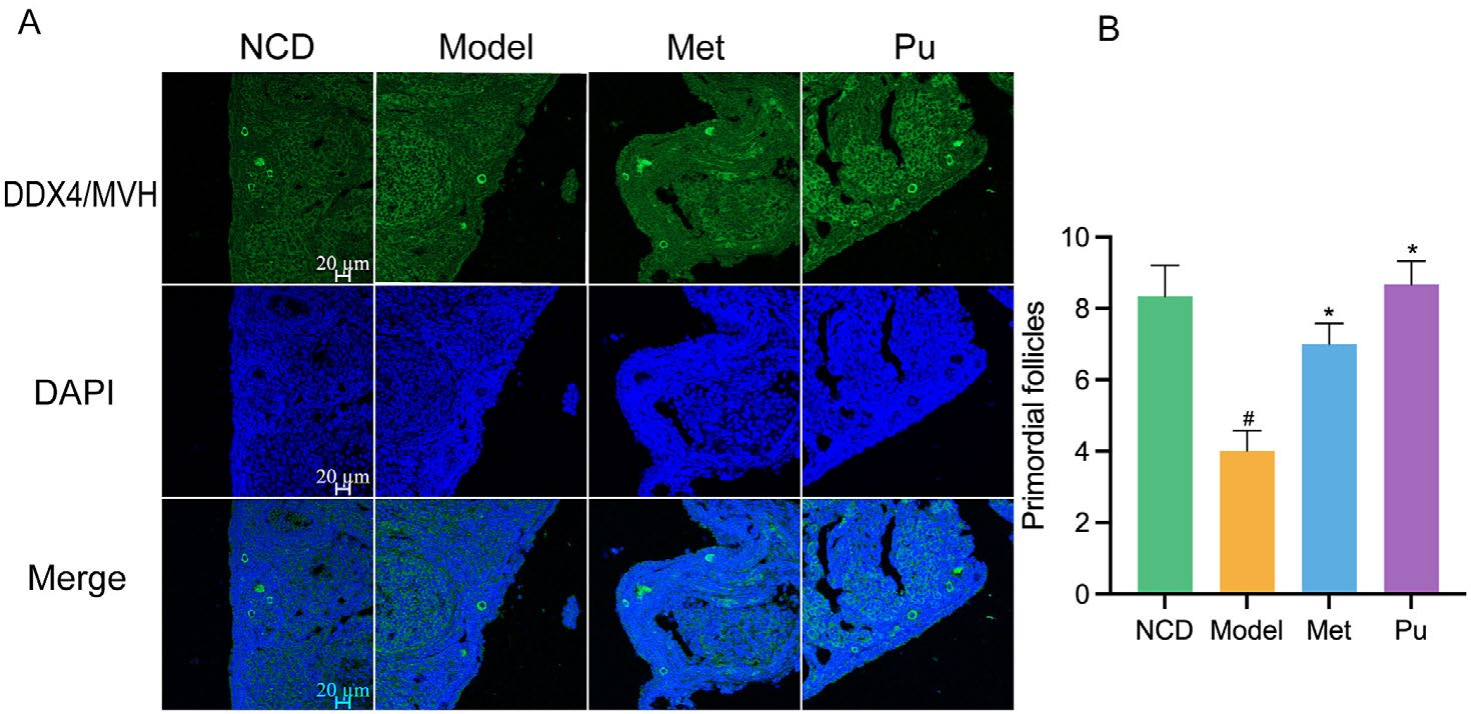
Sea buckthorn pulp affects the expression of germ cell markers in PCOS mice. (A) Immunofluorescence staining of DDX4/Mvh in ovaries of mice from different groups. Scale bar: 20 μm. (B) Number of primordial follicles in different groups. Data are presented as mean ± SEM, with *n* = 6 per group. The symbol # indicates a statistically significant difference compared to the normal control group (*p* < 0.05), and the symbol * indicates a statistically significant difference compared to the model group (*p* < 0.05).

To investigate the effect of sea buckthorn pulp on ovarian germ cell function, we examined the expression of the germ cell‐specific marker DDX4/Mvh, an ATP‐dependent RNA helicase essential for germ cell development (Noyes et al. 2023). Immunofluorescence staining (Figure 6A) revealed that, compared with the control group, the DDX4‐positive signal in the ovaries of the PCOS model group was significantly weakened (Figure 6B). This suggests that the primordial germ cell pool or early follicular reserve in the ovaries may be impaired under PCOS conditions. Importantly, sea buckthorn pulp treatment effectively reversed this trend, leading to a significant recovery of DDX4 expression levels in the ovaries (Figure 6B). This result reveals for the first time that sea buckthorn pulp not only improves the structure of formed follicles but also promotes the expression of germ cell markers within the ovary. This implies that its therapeutic effect may extend to the protection and support of the primordial follicle pool or oogonia, thereby improving ovarian reproductive potential at an earlier, more upstream stage.

### 3.6 Network Pharmacology Predicts the Signaling Pathways Through Which Sea Buckthorn Improves PCOS

**FIGURE 7:**
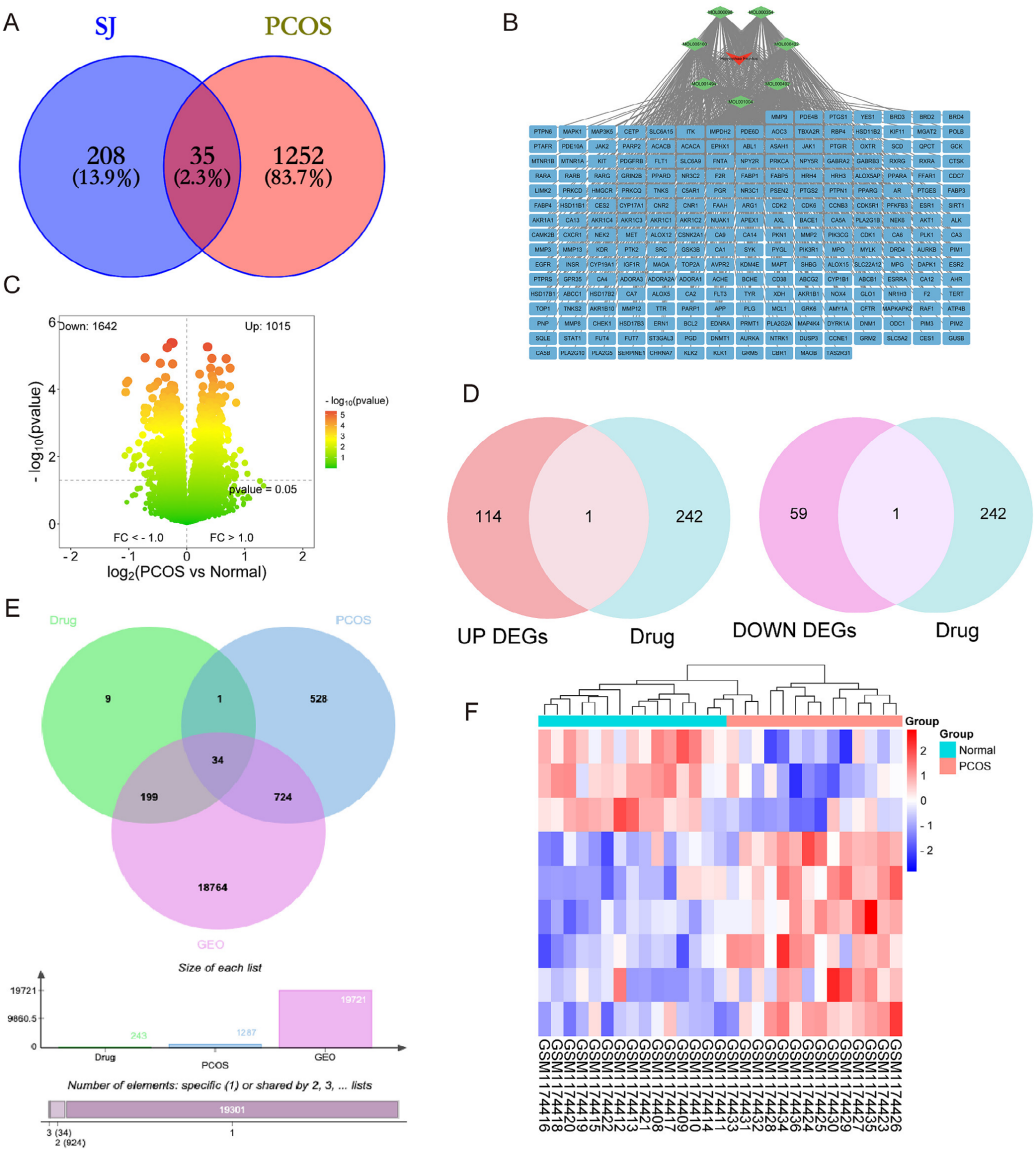
Targets of SJ active components and differentially expressed genes in PCOS. (A) Venn diagram of the intersection between drug component targets and disease targets, with blue‐purple representing the drug and pink representing the disease; (B) Network diagram of active components and their targets. Blue represents all active component targets, green represents the active components of seabuckthorn, and red represents seabuckthorn itself; (C) Volcano plot of differentially expressed genes in the GSE48301 dataset; (D) Intersection of upregulated and downregulated genes in PCOS with SJ targets. Blue represents drug target genes, and pink represents upregulated and downregulated genes respectively; (E) Interaction diagram of GEO, SJ, and PCOS targets. Blue and pink represent PCOS genes and the GEO dataset, and green represents drug targets; (F) Heatmap of overlapping targets. Pink and blue represent the disease group and normal group, respectively.

To further analyze the potential mechanism of sea buckthorn seed oil in improving polycystic ovary syndrome (PCOS), we employed network pharmacology. First, we screened for effective targets of SJ and PCOS in TCMSP, identifying 33 active components of SJ, among which seven could serve as effective active ingredients, corresponding to 247 targets. Using Cytoscape, we constructed an SJ active component‐target network, where blue represents targets and green represents effective components of SJ (Figure 7B). PCOS targets were retrieved from the GeneCards, OMIM, and DisGeNET databases, resulting in 1387 unique PCOS targets after deduplication. A Venn diagram of the intersection between PCOS and SJ was plotted using the Venny 2.1.0 website (Figure 7A). We analyzed datasets screened from the GEO database, identifying 1642 downregulated genes and 1015 upregulated genes (Figure 7C). The intersection of upregulated genes with SJ targets and downregulated genes with SJ targets was subsequently examined (Figure 7D). A Venn diagram generated on the online bioinformatics platform indicated 243 SJ targets, 1287 PCOS genes, and 19,721 GEO database genes. The intersection of all three datasets yielded 34 targets (Figure 7E). Analysis of GEO data resulted in a top 40 heatmap (Figure 7F).

**FIGURE 8:**
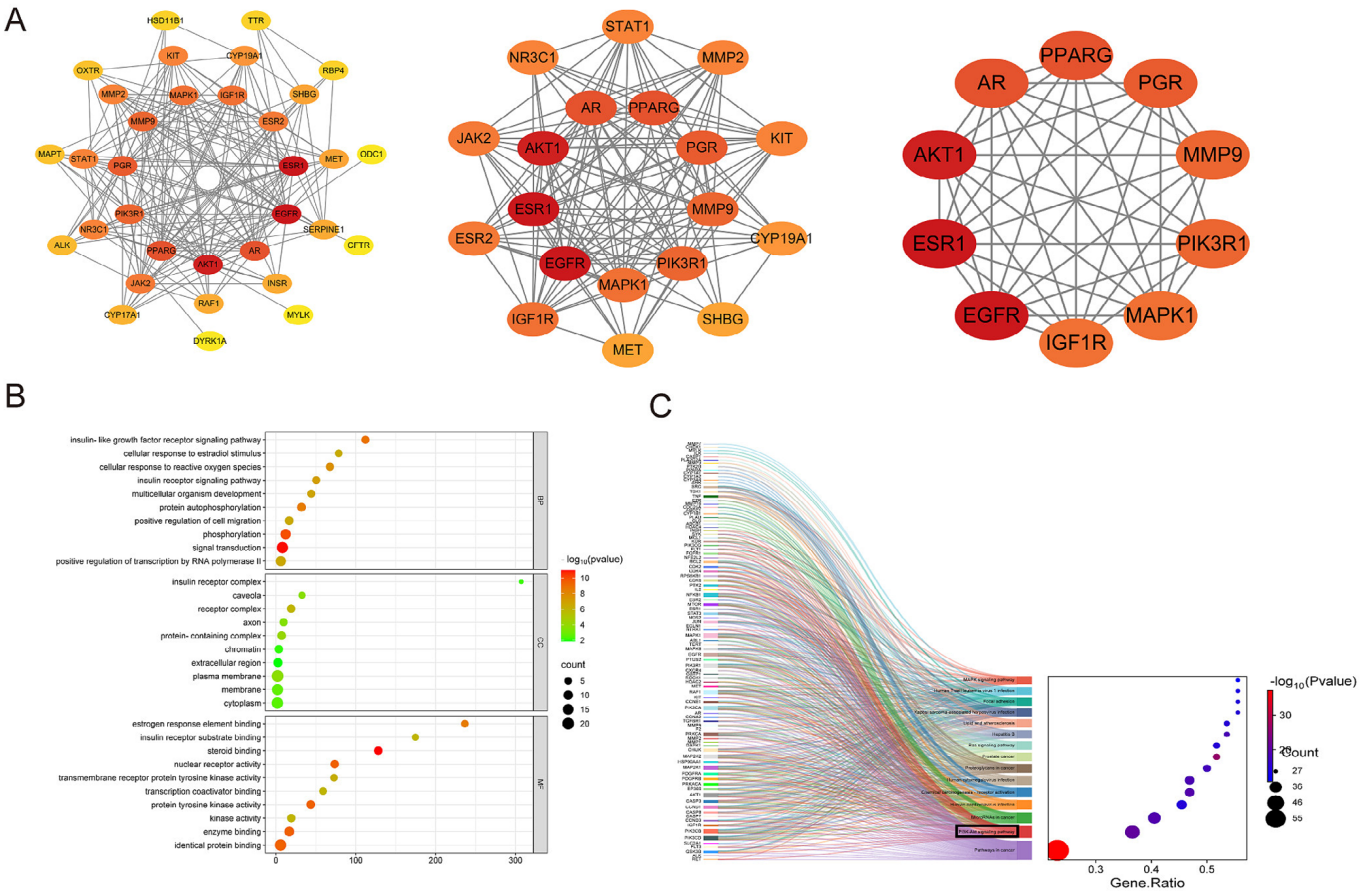
Screening and Enrichment Analysis of Core Targets. (A) Selection of core targets in the PPI network; the larger the circle and the darker the color, the higher the importance in the network. (B) GO enrichment analysis; (C) Top 15 KEGG Sankey diagram. The left side represents the intersecting targets, the middle represents the pathways, and the curves indicate their correlations. The bubble chart on the far right shows the KEGG results, where larger and redder circles indicate more significant enriched pathways.

To further study SJ treatment for polycystic ovary syndrome, Cytoscape software was used to analyze and screen 34 targets (Figure 8A). With Degree > 15, 10 potential targets that may be the most important core targets for treating polycystic ovary syndrome were identified, including AKT1, ESR1, EGFR, IGF1R, MAPK1, PIK3R1, MMP9, PGR, PPARG, and AR. We selected Degree > 15 as a criterion for assessing the importance of the targets.

GO and KEGG enrichment analyses were conducted on 35 targets to perform GO analysis and study how biological information can be used to explore the progress in the treatment and development of PCOS. In the GO analysis, the larger the circle, the more targets were enriched in that entry (Figure 8B). Gene Ontology (GO) analysis revealed distinct enrichment patterns for the 35 targets. In the biological process (BP) category, terms were predominantly enriched in processes such as positive regulation of RNA polymerase II transcription, signal transduction, phosphorylation, positive regulation of cell migration, protein phosphorylation, and multicellular organism development. For cellular components (CC), the targets were mainly localized to the cytoplasm, cell membrane, plasma membrane, extracellular region, chromatin, and protein complexes. Regarding molecular functions (MF), significant enrichment was observed in identical protein binding, enzyme binding, kinase activity, protein tyrosine kinase activity, transcription coactivator binding, and transmembrane receptor protein tyrosine kinase activity. KEGG enrichment analysis was performed to explore how SJ exerts a therapeutic effect on PCOS. As shown in Figure 8C, the top 15 pathways enriched across all pathways are presented, with the ‘PI3K‐AKT signaling pathway’ ranking second.

### 3.7 Sea Buckthorn Pulp May Improve Polycystic Ovary Syndrome by Promoting the PI3K/AKT/mTOR Signaling Pathway

**FIGURE 9:**
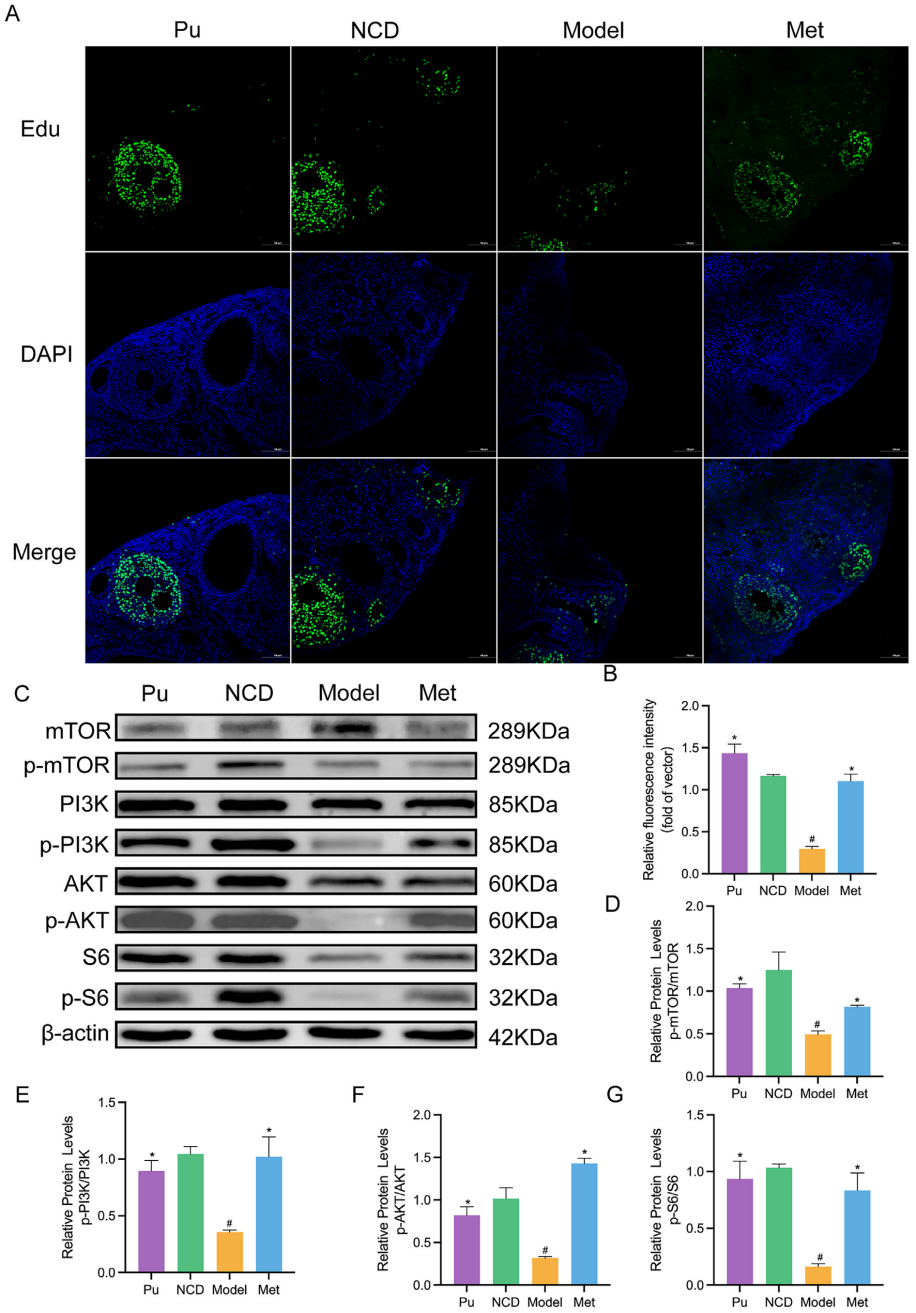
Sea buckthorn pulp improves ovarian proliferative function and upregulates the phosphorylation of PI3K/AKT/mTOR pathway proteins in the ovaries of PCOS mice. (A) EdU staining images were used to analyze ovarian proliferative function. Scale bar: 100 μm. (B) Quantitative analysis of relative fluorescence intensity. (C) Western blot analysis of mTOR, PI3K, and AKT protein expression in ovarian tissues from each group. (D–G) Relative expression levels of PI3K, AKT, mTOR, and S6 proteins in the ovaries of different groups. Data are presented as mean ± SEM, with *n* = 6 per group. The symbol # indicates a statistically significant difference compared to the normal control group (*p* < 0.05), and the symbol * indicates a statistically significant difference compared to the model group (*p* < 0.05).

To investigate the specific mechanism by which sea buckthorn pulp improves ovarian function in PCOS, we first evaluated its effect on ovarian cell proliferation. EdU staining results showed that the ovarian cell proliferation capacity was significantly reduced in the PCOS model group. After treatment with sea buckthorn pulp, ovarian proliferative activity was markedly enhanced, an effect that was even superior to that of the positive control drug, metformin (Figure 9A,B). This indicates that sea buckthorn pulp has the potential to directly promote ovarian cell proliferation.

To explore the underlying molecular mechanism, we focused on the PI3K/Akt/mTOR signaling pathway, a key pathway regulating cell growth and proliferation (Qiu et al. 2020). The PI3K signaling pathway is a fundamental pathway regulating cell proliferation, survival, migration, and metabolism, and is involved in various physiological and pathological processes (Liu et al. 2021). Recent studies have shown that the PI3K/Akt signaling pathway serves as a key regulator of granulosa cell growth and apoptosis during ovarian follicular development (Gong et al. 2020). Western blot analysis demonstrated that the phosphorylation levels of key proteins in this pathway (PI3K, AKT, mTOR, S6) were generally decreased in ovarian tissue of PCOS mice, suggesting pathway inhibition. Sea buckthorn pulp treatment significantly reversed this inhibited state, upregulating the levels of p‐PI3K, p‐AKT, p‐mTOR, and p‐S6 (Figure 9C–G). Notably, the activation of this pathway occurred concomitantly with the therapeutic effects, including improved insulin sensitivity indices, decreased serum androgen levels, and restored follicular development.

## 4 Conclusion

Polycystic ovary syndrome (PCOS) is a common metabolic disorder among women of reproductive age, characterized by insulin resistance, hyperandrogenemia, chronic inflammation, oxidative stress, and other metabolic abnormalities, which also lead to ovulatory dysfunction and ovarian dysfunction (Jiang et al. 2022; Zhang et al. 2023). In clinical practice, due to its complex and still incompletely understood etiology and pathogenesis, initial treatment primarily focuses on symptom relief (Peng et al. 2023). Traditional Chinese Medicine (TCM) posits that the pathogenesis of PCOS is primarily attributed to long‐term spleen deficiency and phlegm‐dampness (Hu et al. 2025). The disease is closely related to the dysfunction of the Kidney, Spleen, and Liver, with a principal emphasis on Kidney deficiency and Spleen deficiency (Lyu et al. 2025). These deficiencies, compounded by the accumulation of pathological factors such as phlegm‐dampness and blood stasis, disrupt the functional axis of the “Kidney–Tiangui–Chong–Ren–Uterus” (the TCM reproductive axis), thereby leading to the manifestation of the disease (Qian et al. 2021).

Sea buckthorn (*Hippophae rhamnoides L*.), a medicinal and edible herb, holds substantial potential for application. In Traditional Chinese Medicine (TCM), sea buckthorn is characterized as warm in nature, with sour and astringent flavors. The sour flavor possesses astringent and tonifying properties, while the astringent flavor consolidates and secures (Liu et al. 2026). Its warm nature, without being drying, allows it to disperse cold, warm and unblock, and warm and transform—precisely targeting the characteristics of phlegm‐dampness as a sticky, lingering pathogenic factor and the pattern of internal accumulation of cold‐dampness (Liu et al. 2026). It can warm‐transform cold‐dampness and dredge the collaterals without the drawback of drying warmth injuring yin. In terms of meridian tropism, sea buckthorn enters the Spleen and Stomach meridians, directly addressing the core disease location of PCOS—“Spleen deficiency leading to impaired transportation and transformation, and endogenous phlegm‐dampness.” It fortifies the Spleen, boosts Qi, harmonizes the Stomach, and promotes digestion, thereby restoring the transporting and transforming functions of the Spleen and Stomach and cutting off the source of phlegm‐dampness production, embodying the TCM principle of “treating disease by addressing its root cause.” Regarding its therapeutic effects, its action of “dispelling phlegm” refers to resolving phlegm, dispelling dampness, eliminating turbidity, and dispersing nodules. It can remove the congested phlegm‐dampness in the lower Jiao and dredge the uterine vessels. Its effect of “activating blood and dispersing stasis” can unblock Qi and blood stasis in the Chong and Ren vessels and resolve the aggregations and accumulations formed by phlegm‐dampness and blood stasis. It integrates the multiple effects of resolving phlegm, dispelling dampness, activating blood, and fortifying the Spleen, perfectly aligning with the core treatment principles for this disease: “fortifying the Spleen to resolve phlegm, dispelling dampness to unblock the collaterals, and regulating the Chong and Ren vessels.”

Sea buckthorn has a history of over 1300 years as a medicinal herb, used for treating conditions such as cough, asthma, indigestion, and peptic ulcers (Zheng et al. 2025). Sea buckthorn pulp is rich in vitamins, polyphenols, and other unstable active components (Ma et al. 2022; Wang, Zhao, et al. 2022), making it an important resource for both medicinal and dietary purposes. Studies have shown that sea buckthorn pulp can treat citric acid‐induced cough in guinea pigs and effectively promote phenol red secretion, indicating its antitussive and expectorant effects (Chen et al. 2024; Ouyang et al. 2024). However, no study to date has demonstrated the potential intervention effects of sea buckthorn pulp on PCOS.

The findings of this study indicate that sea buckthorn pulp can ameliorate polycystic ovary syndrome (PCOS) in mice, restore ovarian function, and thereby improve reproductive outcomes. This therapeutic effect may be attributed to the traditional Chinese medicinal properties of sea buckthorn, namely its ability to resolve phlegm, dispel dampness, unblock collaterals, and regulate menstruation. In the present study, we demonstrated that sea buckthorn pulp can mitigate hyperandrogenism‐induced PCOS and reduce its detrimental effects on primordial follicles, consequently improving ovarian function. Utilizing network pharmacology analysis, we predicted the active components of sea buckthorn, their potential targets, and disease‐related targets associated with PCOS. The strategy of intersecting the predicted targets of sea buckthorn with polycystic ovary syndrome (PCOS)‐related targets and differentially expressed genes (DEGs) from a specific dataset (GSE48301) was designed to achieve a multi‐layered screening process. Through GO and KEGG enrichment analyses, the PI3K/Akt/mTOR signaling pathway was identified as playing a crucial role in ovarian follicular development. Furthermore, we experimentally validated that the therapeutic effect of sea buckthorn pulp on PCOS may be associated with the modulation of the PI3K/AKT/mTOR signaling pathway.

In this study, a PCOS model characterized by obesity and insulin resistance was successfully established in mice through a combination of a high‐fat diet and letrozole administration (Qiu et al. 2020). The model exhibited obesity, elevated blood glucose, glucose intolerance, insulin resistance, disrupted estrous cycles, impaired ovarian morphology, reduced ovulation, and abnormal steroid hormone levels (Stener‐Victorin et al. 2024). Collectively, these parameters suggested the successful establishment of a PCOS‐like phenotype and associated metabolic disturbances in the model mice. Both sea buckthorn pulp and metformin treatments were associated with improvements in metabolic parameters, including body shape, body weight, and fasting blood glucose levels. However, metformin was less effective than sea buckthorn pulp in enhancing ovarian proliferation in PCOS mice. Moreover, compared to metformin, sea buckthorn pulp, as a natural product, offers the advantage of multi‐target intervention.

To assess ovarian proliferative function, we employed EdU staining, and the findings were consistent with the aforementioned results. The PI3K/Akt/mTOR signaling pathway plays a crucial role in ovarian follicular development. The PI3K signaling transduction pathway is a key regulator of cell proliferation, growth, and differentiation (Tan et al. 2022) and exerts close and critical regulatory control over follicular differentiation, growth, and survival. Akt plays a pivotal role in ovarian development, influencing granulosa cell proliferation and folliculogenesis (Qiu et al. 2020). When PI3K is activated, it phosphorylates PIP2 to generate PIP3, which in turn phosphorylates Akt (Liang et al. 2024); activated Akt can then stimulate mTOR. Activation of this pathway promotes the proliferation and secretory function of granulosa cells, thereby influencing follicular development (Li et al. 2021). Our Western blot results showed that sea buckthorn pulp upregulated the phosphorylation of PI3K/AKT/mTOR in ovarian tissue. Although this study did not directly isolate specific ovarian cell types (e.g., granulosa cells), this finding suggests an overall improvement in the ovarian signaling pathway. Studies have indicated that the PI3K/AKT/mTOR pathway is a key negative regulator of autophagy in granulosa cells (Gong et al. 2020; Liu et al. 2021). Given that granulosa cells are the most abundant somatic cells in the ovary, we speculate that the therapeutic effect of sea buckthorn pulp may be mediated, at least in part, through the modulation of granulosa cell autophagy via this pathway. This provides a potential cellular mechanism for the observed histological improvement in follicular development. Of course, this specific mechanism requires further validation through future in vitro experiments using granulosa cells. This experiment focused solely on sea buckthorn pulp as a substance. As a natural mixture, the specific active components of sea buckthorn pulp (such as flavonoids, vitamins, fatty acids, etc.) have not been fully characterized, and it remains unclear whether the therapeutic efficacy stems from a single component or the synergistic action of multiple constituents. Future studies are needed to further elucidate these aspects.

It is worth noting that the role of autophagy in PCOS is bidirectional, with abnormalities manifesting as either excessive activation or suppression (Chen et al. 2021; Liu et al. 2021). Therefore, the regulation of the PI3K/Akt/mTOR pathway by sea buckthorn pulp may not simply promote or inhibit autophagy, but rather maintain its dynamic equilibrium. In the present study, the PCOS model induced by letrozole combined with a high‐fat diet exhibited decreased PI3K/Akt/mTOR pathway activity, whereas intervention with sea buckthorn pulp reactivated this pathway, accompanied by improvements in ovarian morphology and the estrous cycle. Thus, we speculate that, under the conditions of this model, sea buckthorn pulp exerts a protective effect by restoring PI3K/Akt/mTOR signaling activity, thereby maintaining autophagic homeostasis in granulosa cells.

Our study has several limitations. First, the investigation relied on a classic PCOS mouse model induced by letrozole combined with a high‐fat diet. Although this model reliably reproduces core features of PCOS, including hyperandrogenemia, ovulatory dysfunction, and polycystic ovarian morphology, its pathophysiology does not fully mirror the complexity and chronic progression of metabolic disturbances seen in human PCOS. Future studies incorporating clinical cohorts are needed to address this translational gap. Second, the bioactive composition of sea buckthorn pulp—including flavonoids, vitamins, and fatty acids—varies with plant variety, geographical origin, harvest time, and processing methods. While we used a single, standardized batch to ensure consistency within the treatment group, such natural variability may affect the reproducibility of results in follow‐up studies using different sources. Third, the beneficial effects on hormonal and metabolic parameters demonstrated here are confined to the experimental model. The safety, optimal dosing regimen, and efficacy of sea buckthorn berry pulp in women with PCOS remain to be established. Well‐designed clinical trials are the essential next step to evaluate its translational potential. Fourth, although we identified changes in the PI3K/AKT/mTOR pathway, the study lacks functional validation (e.g., using pharmacological inhibitors or genetic approaches) to establish a causal link between pathway modulation and the observed phenotypic improvements. Future studies employing such tools are needed to confirm the mechanistic role of this pathway. Furthermore, the network pharmacology approach used to predict potential targets of sea buckthorn and to identify core targets by intersecting with PCOS‐related genes has inherent constraints. Although the GSE48301 dataset is a valuable and widely cited resource in PCOS research, the differentially expressed genes derived from it are influenced by the specific experimental conditions, model system, and sample size of the original study. Consequently, the identified “core targets” may not be fully generalizable across all PCOS subtypes. Finally, this study focused primarily on hormonal, morphological, and gluco‐metabolic outcomes, leaving more integrated mechanistic insights—such as those involving metabolomic shifts or gut microbiota modulation—unexplored.

Despite these limitations, our findings possess clear translational relevance. The observed improvements in hyperandrogenism, insulin resistance, and inflammation align with the increasing recognition that targeting metabolic and inflammatory pathways is crucial for comprehensive PCOS management (Ercan et al. 2025; Karateke et al. 2018). The fact that sea buckthorn, a dietary supplement, positively modulated these key pathological pathways underscores its potential as a complementary or alternative strategy. Future research should prioritize human studies to validate these preclinical benefits, while also investigating the specific bioactive compounds responsible for the effects to enable standardization for clinical use.

In summary, the work in this study reveals that sea buckthorn pulp can ameliorate polycystic ovary syndrome (PCOS), and its mechanism may be associated with the enhancement of ovarian proliferative function. Whether sea buckthorn pulp directly inhibits the activation of the primordial follicle pool or mitigates damage to growing follicles, thereby preventing excessive recruitment of primordial follicles into the growing pool and preserving the ovarian reserve, requires further investigation. We hope that this study will provide new insights for research and therapeutic strategies in fertility preservation.

## Author Contributions

**Han Xiaoya:** investigation, validation, methodology, writing – original draft. **Yang Yuanyuan:** software, methodology. **Liu Hetao:** methodology, investigation,clinical guideline. **Ma Cunling:** methodology, software. **Tian Xiaofan:** methodology, investigation. **He Rui:** writing – review and editing, methodology, conceptualization, funding acquisition, validation, supervision. **Chen Xiaojiang:** software, writing – review and editing, validation. **Peng Qingjie:** investigation, software. **Li Na:** investigation, methodology. **Wu Xin:** methodology, clinical guideline.

## Funding

This work was supported by the National Natural Science Foundation of China (82201000), the Natural Science Foundation of Ningxia Province (2022AAC02033), the Ningxia Key R&D Program Projects (2021BEB04034, 2022BEG02034), the Ningxia Medical University University‐level Research Project (XJKF240321), the Ningxia Science and Technology Innovation Leading Talent Training Project (2020GKLRLX11), and the Ningxia Science and Technology Innovation Team (2025CXTD003).

## Conflicts of Interest

The authors declare no conflicts of interest.

## Supporting information


**Table S1:** Representative metabolites identified in sea buckthorn pulp using UPLC–MS/MS‐based metabolomic analysis.

## Data Availability

The data that support the findings of this study are available from the corresponding author upon reasonable request.
