# Supplementary material for: Study on the Effects and Mechanisms of Sea Buckthorn Fruit Pulp in Improving Polycystic Ovary Syndrome
Source: Food Sci Nutr. 2026 Aug 12;14(8):e72181. doi: 10.1002/fsn3.72181 (PMC13463749; doi:10.1002/fsn3.72181)
Supplement: Supplementary file 1 [file FSN3-14-e72181-s001.docx]

Supplementary Table S1. Representative metabolites identified in sea buckthorn pulp using UPLC–MS/MS-based metabolomic analysis.

| **Index** | **Compound** | **Molecular Formula** |
| --- | --- | --- |
| Lmsp004045 | Isorhamnetin-3-O-glucoside-7-O-rhamnoside | C_28_H_32_O_16_ |
| pme1540 | Isorhamnetin-3-O-neohesperidoside | C_28_H_32_O_16_ |
| mws0045 | Quercetin-3-O-rhamnoside(Quercitrin) | C_21_H_20_O_11_ |
| Lmsp004247 | Isorhamnetin-3-O-arabinoside-7-O-rhamnoside | C_27_H_30_O_15_ |
| Lmdp003171 | Azalein (Azaleatin-3-O-rhamnoside) | C_22_H_22_O_11_ |
| HJAP127 | Quercetin-3-O-(2''-O-Rhamnosyl)rutinoside | C_33_H_40_O_20_ |
| Lmsp004749 | Syringetin-3-O-rutinoside | C_29_H_34_O_17_ |
| mws0120 | Choline Alfoscerate | C_8_H_20_NO_6_P |
| Zmgp002857 | quercetin 3-O-α-rhamnopyranosyl (1→2)-[α-rhamnopyranosyl (1→6)]-β-glucopyranoside | C_33_H_40_O_20_ |
| HJAP128 | Isorhamnetin-3-O-rutinoside-7-O-rhamnoside | C_34_H_42_O_20_ |
| Lmmn006022 | Rhamnetin-3-O-rhamnoside | C_22_H_22_O_11_ |
| Hmgp003409 | Quercetin-3-O-Soph-7-O-Rha b | C_33_H_40_O_20_ |
| Zmgp004436 | okanin 4'-(6''-O-acetylglucoside) | C_23_H_24_O_13_ |
| HJAP119 | Rhamnetin-3-O-Rutinoside-5-O-rhamnoside | C_34_H_42_O_20_ |
| Lmsp003252 | Isorhamnetin-3-O-sophoroside | C_28_H_32_O_17_ |
| pmn001695 | Trihydroxycinnamoylquinic acid | C_16_H_20_O_10_ |
| Lmmp002334 | Quercetin-3-O-rutinoside-7-O-glucoside | C_33_H_40_O_21_ |
| Lmsp003939 | Isorhamnetin-3-O-galactoide-7-O-rhamnoside | C_28_H_32_O_16_ |
| mws0066 | Isorhamnetin | C_16_H_12_O_7_ |

Note: Twenty representative metabolites identified in sea buckthorn pulp are presented.
